# Efficient and simple generation of multiple unmarked gene deletions in *Mycobacterium smegmatis*

**DOI:** 10.1038/srep22922

**Published:** 2016-03-14

**Authors:** Xu-Jian Mao, Mei-Yi Yan, Hui Zhu, Xiao-Peng Guo, Yi-Cheng Sun

**Affiliations:** 1 MOH Key Laboratory of Systems Biology of Pathogens, Institute of Pathogen Biology, and Center for Tuberculosis Research, Chinese Academy of Medical Sciences and Peking Union Medical College, Beijing, P.R. China

## Abstract

Research on mycobacterial genetics relies heavily on techniques for directed gene mutation, but genetic studies are often hampered by the difficulty of generating gene deletions in mycobacteria. We developed an efficient and improved deletion system, described here in detail, which can be used to construct multiple unmarked recombinants in mycobacteria. We tested this system by using it to sequentially delete four pairs of toxin-antitoxin genes in *Mycobacterium smegmatis*.

Genus *Mycobacterium* includes the pathogenic species *tuberculosis* and *leprae*, as well as many other opportunistic pathogens that occupy specific environmental niches. Both scientific study and medical control of mycobacteria require efficient molecular tools for various types of genetic manipulation, especially directed mutagenesis and gene deletion^1^. The development and application of such tools will accelerate study of the mechanisms underlying pathogenesis, drug action, and drug resistance in mycobacteria.

A wide range of genetic manipulations using suicide plasmids, non-replicating plasmids, specialized transduction with mycobacteriophages, counter-selectable markers, and long linear DNA fragments have been developed to obtain mycobacterial mutants^2^,^3^,^4^,^5^,^6^. However, these methods typically yield small numbers of mutants and high background levels of false-positive colonies^7^. To overcome this challenge, a plasmid (pJV53) containing recombination proteins gp60 and gp61 of mycobacteriophage Che9c has been used to increase the efficiency of homologous recombination in both *Mycobacterium smegmatis* and *Mycobacterium tuberculosis*^8^. The phage recombinases encoded by pJV53 catalyze homologous recombination of linear DNA into a desired chromosomal locus, and the antibiotic resistance cassette can be used to isolate recombinants. However, only a few antibiotic genes are available for use in mycobacteria, hampering functional studies of redundant genes in these organisms. For example, *M. tuberculosis* has at least 79 genes encoding potential toxin-antitoxin pairs^9^. Because mutation of any one of these genes might be compensated by other genes with similar functions, elucidation of their roles will require multiple gene deletion or complementation experiments. One way to solve this problem would be to construct unmarked mutations, which can be obtained by two-step homologous recombination including selection and counter-selection of the individual allelic exchange events using both positive and negative markers^10^, or by a different method using a site-specific recombinase or resolvase^4^,^11^,^12^.

Recently, a new sequence-specific system based on the endogenous Xer recombinase (Xer-cise) was successfully used in *Escherichia coli* and *Mycobacteria*^13^,^14^,^15^. In this simple and practical system, the antibiotic resistance gene flanked by *dif* sites is recognized and removed by the endogenous recombinases XerC and XerD^16^. However, the antibiotic resistance gene used in this system is the *hyg* cassette, which prevents its use if the target strain is already Hyg-resistant. In addition, it remains unclear whether and how this system could be used for multiple gene deletions.

In this report, we modified pJV53 by introducing a *gfp* gene to facilitate screening for loss of the helper plasmid following recombination. We also created *dif*-flanked Zeo- and Hyg-resistance cassettes containing multiple restriction enzyme sites. We tested this system and demonstrated that it can be used for sequential deletion of multiple genes in *M. smegmatis*.

## Results

### Generation of plasmids for the recombination system

**Figure 1:**
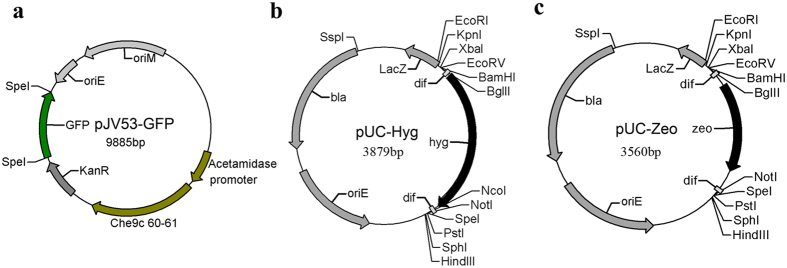
Gene knock out system in *M. smegmatis*. (**a**) The plasmid pJV53-GFP (GenBank KU306402) is an inducible expression vector for gp60 and gp61 and expresses the *gfp* gene. (**b**) pUC-Hyg (GenBank KU306403) is a cloning vector for the homologous arms of the target gene. The flanking cassette includes multiple restriction enzyme sites for the cloning of homologous regions upstream and downstream of the *dif-hyg-dif* cassette. (**c**) pUC-Zeo (GenBank KU306404) is a cloning vector for homologous arms of the target gene. The flanking cassette includes multiple restriction enzyme sites for the cloning of homologous regions upstream and downstream of the *dif-zeo-dif* cassette.

**Figure 2:**
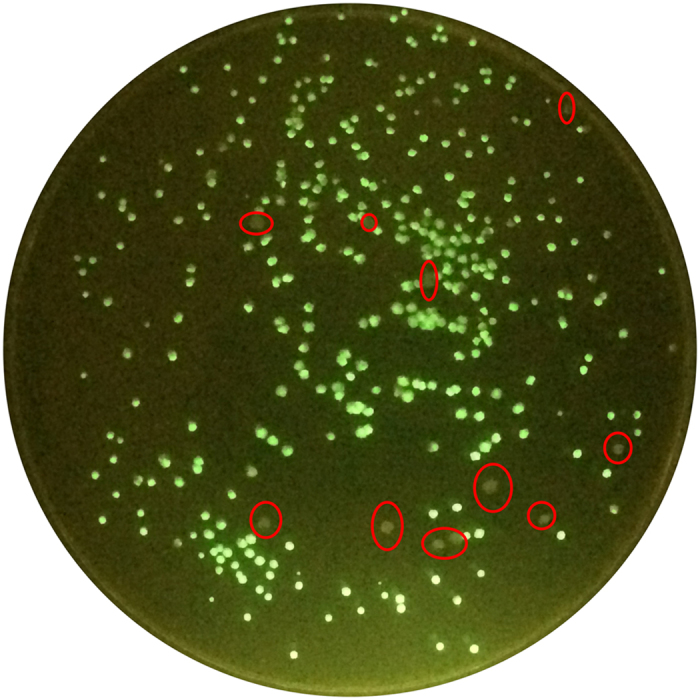
Identification of *M. smegmatis* carrying pJV53-GFP. Pictures were taken under illumination with a Nightsea Bluestar flashlight. Colonies with GFP signals still carry the plasmid pJV53-GFP whereas colonies without GFP signals (in red circles) have lost the plasmid pJV53-GFP.

The helper plasmid pJV53, which expresses the Che9c genes, is widely used for gene recombination^8^. Following recombination, cells transformed with pJV53 are plated on sucrose-containing media to cure the plasmid. Because it is sometimes difficult to cure a plasmid from recombinant cells, pJV53 was engineered to have a temperature-sensitive origin of replication, which facilitates elimination of the plasmid at high temperature (42 °C)^12^,^15^. Consequently, mycobacteria harboring this plasmid must grow at low temperature (30 °C), which reduces their growth rate. To overcome this problem, we modified pJV53 by introducing a *gfp* gene (Fig. 1a); the resultant plasmid was designated pJV53-GFP. When *M. smegmatis* harboring pJV53-GFP was plated on 7H10 agar containing 10% sucrose (7H10 + sucrose), cells cured of the plasmid could be easily identified using a NightSea flashlight and filter glasses (Fig. 2). Approximately 6.0% of colonies lost the GFP signal when they were grown on 7H10 + sucrose. Ten colonies without GFP signal were picked; all of them had lost kanamycin resistance, indicating loss of pJV53-GFP.

Next, we constructed improved excisable Hyg- and Zeo-resistance cassettes. Specifically, we cloned the Hyg- or Zeo-resistance gene flanked with *dif* sequences and multiple restriction sites into pUC57, yielding pUC-Hyg or pUC-Zeo, respectively (Fig. 1b,c). The multiple restriction sites facilitate the insertion of homologous genes, and the Zeo-resistance cassette will broaden the applicability of this recombination system. In addition, we designed the sequence around the *dif* sites to allow the creation of in-frame deletions as described previously^15^. To avoid disrupting the expression of downstream genes, it is important to create in-frame deletions in mycobacteria.

### Deletion of single genes in *M. smegmatis*

**Figure 3:**
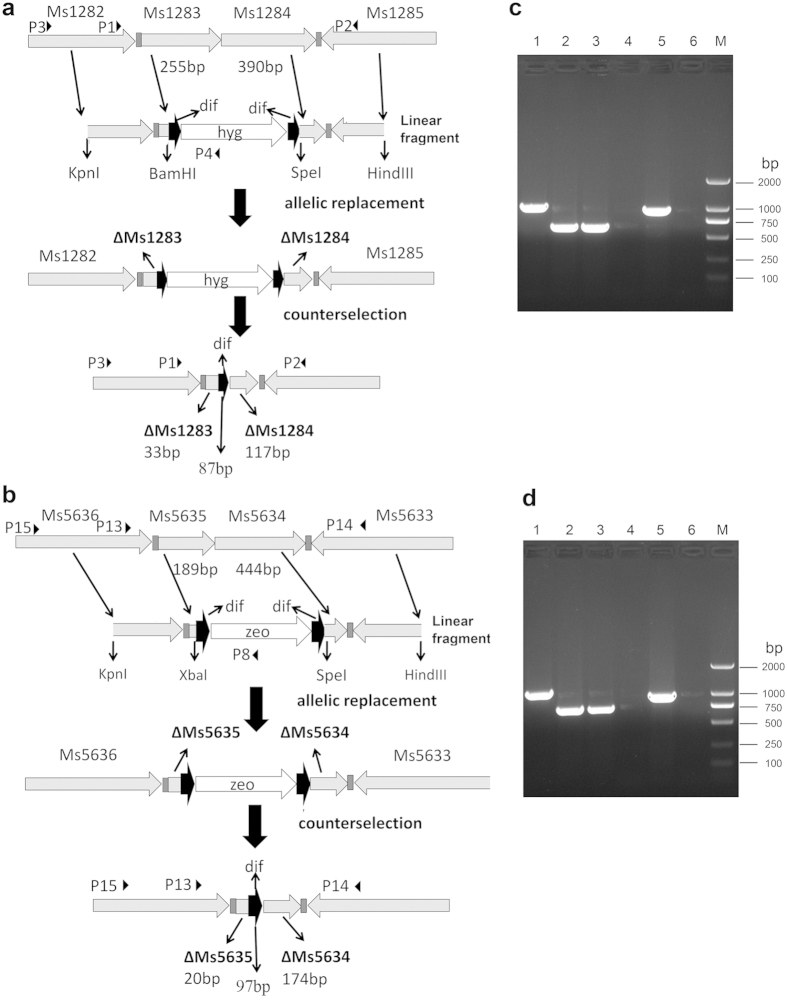
Deletion of Ms1283–1284 and Ms5634–5635 genes in *M. smegmatis*. (**a**) and (**b**) Diagrams of the deletions of Ms1283–1284 and Ms5634–5635 genes. dif indicates the *dif* sequence and its direction. The triangles indicate the primers and their directions. The linear fragments were cut from pYC710 and pYC799, respectively. (**c,d**) Verification of Ms1283–1284 and Ms5634–5635 deletions. Lane M, DNA ladder; lane 1, PCR products from wild type *M. smegmatis* using primer p1/p2 (**c**) or p13/p14 (**d**); lane 2, PCR products from the *M. smegmatis* Ms1283–1284 deletion mutant containing hygromycin using primer p1/p2 (**c**) or from the *M. smegmatis* Ms5634–5635 deletion mutant containing zeocin using primer p13/p14 (**d**); lane 3, PCR products from unmarked *M. smegmatis* Ms1283–1284 deletion mutant using primer p1/p2 or from the unmarked *M. smegmatis* Ms5634–5635 deletion mutant using primer p13/p14 (**d**). lane 4, PCR products from wild type *M. smegmatis* using primer p3/p4 (**c**) or p15/p18 (**d**); lane 5, PCR products from the *M. smegmatis* Ms1283–1284 deletion mutant containing hygromycin using primer p3/p4 (**c**) or from the *M. smegmatis* Ms5634–5635 deletion mutant containing zeocin using primer p15/p18 (**d**); lane 6, PCR products from the unmarked *M. smegmatis* Ms1283–1284 deletion mutant using primer p3/p4 or from the unmarked *M. smegmatis* Ms5634–5635 deletion mutant using primer p15/p18 (**d**).

We first tested the performance of our recombination system by deleting *M. smegmatis* Ms1283–1284 and Ms5634–5635, two pairs of toxin-antitoxin genes. For this purpose, the upstream and downstream fragments of Ms1283–1284 and Ms5634–5635 were amplified and cloned into pUC-Hyg and pUC-Zeo, respectively, yielding plasmids pYC710 and pYC799 (see details in Materials and Methods). About 100 ng of linear recombineering DNA fragments generated by restriction digestion of the resultant plasmids (Fig. 3a,b) was transformed into competent *M. smegmatis* cells harboring pJV53-GFP, which led to the production of 105 ± 31 Hyg-resistant colonies and 87 ± 23 Zeo- resistant colonies. Dozens of Hyg- or Zeo- resistant colonies were picked and tested by PCR using appropriate primers (Fig. 3a,b), and successful recombination was confirmed in 76% ± 8% of Hyg-resistant colonies, and 83% ± 5% of Zeo-resistant colonies. The predicted PCR product of ~900 bp was obtained from a correct Ms1283–1284 mutant using primers p3 and p4 (Fig. 3c lane 5), whereas no amplification was obtained from the wild-type strain (Fig. 3c lane 4). Likewise, the predicted PCR product of ~1000 bp was obtained from the Ms5634–5635 mutant using primers p8 and p15 (Fig. 3d, lane 45), whereas no amplification was obtained for the wild-type strain (Fig. 3d, lane 4). However, the PCR product amplified from the Ms1283–1284 mutant using primers p1 and p2 was ~600 bp instead of the predicted 1735 bp (Fig. 3c, Lane 2), and the PCR product amplified from the Ms5634–5635 mutant using primers p13 and p14 was ~700 bp instead of the predicted 1416 bp (Fig. 3d, lane 2). This might be because the antibiotic resistance cassette was lost in some cells: when the fragments were amplified by PCR, the shorter fragments were preferentially (or exclusively) amplified. The PCR products were sequenced and the results confirmed that the cassette was lost. In fact, a similar phenomenon was also observed when another Xer-cise system was used to construct gene deletions^15^. The mutants were further plated on 7H10 agar to allow excision of the antibiotic resistance cassette. Nine of ten colonies lost the hygromycin resistance gene, whereas eight of ten colonies lost the zeoxin resistance gene. The mutant strains that lost the antibiotic resistance genes were further analyzed by PCR using appropriate primers. As expected, the PCR product amplified from the Ms1283–1284 mutant using primers p1 and p2 was ~600 bp, and the PCR product amplified from the Ms5634–5635 mutant using primers p13 and p14 was ~600 bp; while no PCR products were amplified from the Ms1283–1284 mutant using primers p3 and p4, and no PCR products were amplified from the Ms1283–1284 mutant using primers p15 and p16. The PCR products were sequenced, and the sequencing results showed that the Ms1283–1284 and Ms5634–5635 mutants harbored the designed unmarked in-frame deletions. The pSL001 plasmid containing *loxP* sites was used as a control to delete the Ms5634–5635 gene. Use of 100 ng of linear recombineering DNA fragments for allelic exchange produced 65 ± 21 recombinant colonies, and correct recombination was confirmed in 62% ± 15% of colonies. Thus, the recombination efficiency of our system is comparable to that of the Cre/loxP gene deletion system described previously^17^. Taken together, these results suggested that our modified system can be successfully used to construct gene deletions in *M. smegmatis*.

### Sequential deletion of toxin-antitoxin genes in *M. smegmatis*

**Figure 4:**
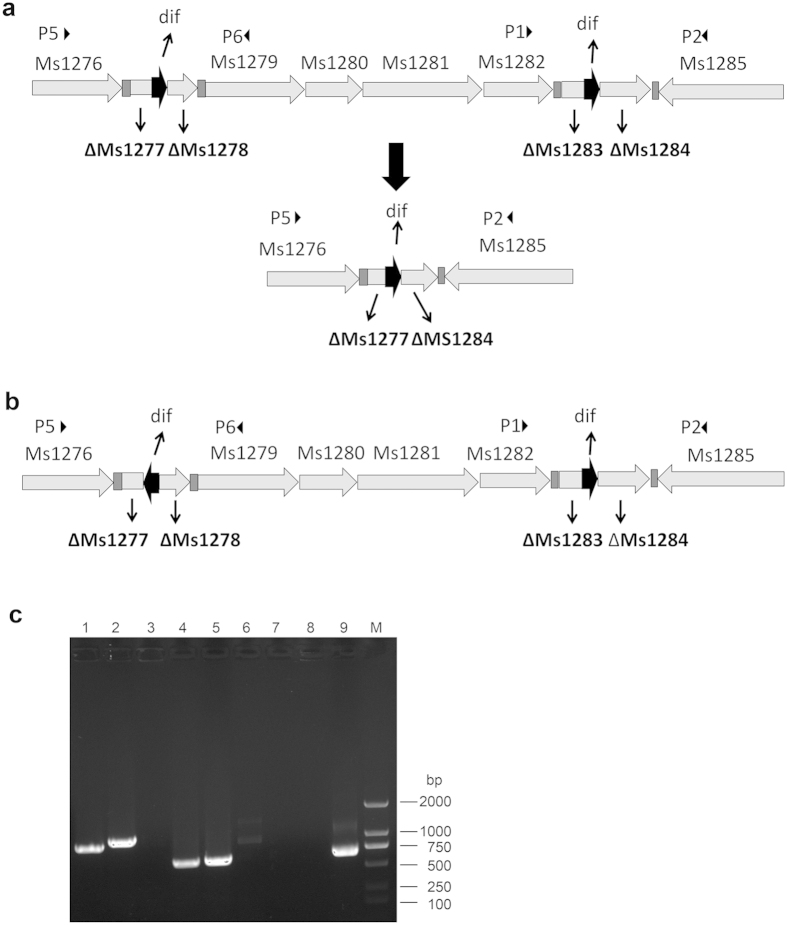
Double deletion of Ms1283–1284 and Ms1277–1278 in *M. smegmatis*. Diagram of the deletion of Ms1283–1284 and Ms1277–1288, in which the *dif* sites are either in the same direction (**a**) or in the opposite direction (**b**). The triangles indicate the primers and their directions. (**c**) Verification of Ms1283–1284 and Ms1277–1278 double deletion mutant. Lane M, DNA ladder; lanes 1–3, PCR products from unmarked wild type *M. smegmatis*, using primer pair p5/p6, p1/p2, and p2/p5; lanes 4–6, PCR products from the Ms1283–1284 and Ms1277–1278 double mutant in which dif sites are in the opposite direction, using the primer pairs p5/p6, p1/p2, and p2/p5; lanes 4–6, PCR products from Ms1283–1284 and Ms1277–1278 double mutants in which *dif* sites are in the same direction, using the primer pairs p5/p6, p1/p2, and p2/p5.

Next, we used our system to construct double deletions in *M. smegmatis*. Ms1277–1278, another pair of toxin-antitoxin genes, is very close to Ms1283–1284 on the chromosome. We hypothesized that the intergenic region between these two pairs of toxin-antitoxin encoding genes would be deleted if the *dif* sites on the plasmids were in the same orientation (Fig. 4a); if the intervening sequence contains essential genes, such deletion would be lethal. Consistent with this hypothesis, no PCR products were obtained from the Ms1277–1278/Ms1283–1284 double mutant using primer pairs p5/p6 and p1/p2 (Fig. 4c, lane 7 and lane 8), whereas the PCR product obtained with primer pairs p2/p5 was ~600 bp, as predicted (Fig. 4a,c, lane 9). When we reversed the orientation of the *dif* sites in the recombineering DNA fragment for deletion of Ms1277–1278, we also obtained the expected PCR products from the Ms1277–1278 and Ms1283–1284 double mutant using primer pairs p1/p2 and p5/p6 (Fig. 4b,c). These results suggested that the cassette could be used for double mutation even when the mutated genes are near each other on the chromosome.

**Figure 5:**
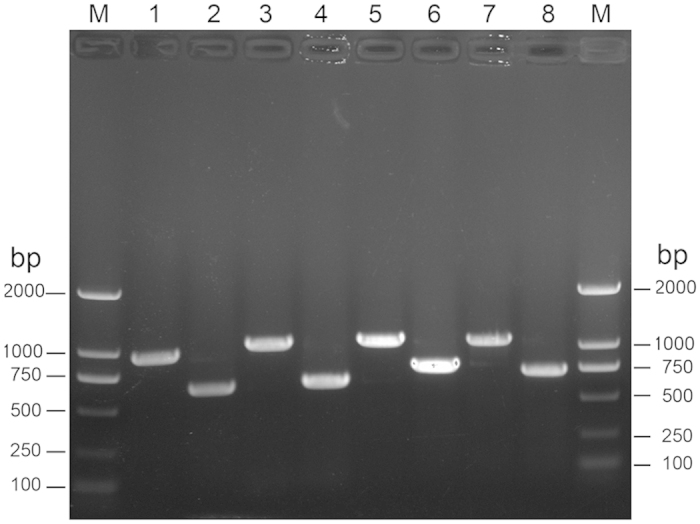
Verification of the Ms1277–1288, Ms1283–1284, Ms4447–4448 and Ms5634–5635 four deletion mutant. Lane M, DNA ladder; PCR products from wild type *M. smegmatis* and the four deletion mutant using p5/p6 (verification of Ms1277–1278 mutation, lanes 1 and 2), and primer p1/p2 (verification of Ms1283–1284 mutation, lanes 3 and 4), and p10/p11 (verification of Ms4447–4448 mutation, lanes 5 and 6), and p13/14 (verification of Ms5634–5645 mutation, lanes 7 and 8).

Next, we investigated whether this system could be used for multiple deletions. *M. smegmatis* has four known pairs of toxin-antitoxin genes^9^: Ms1277–1278, Ms1283–1284, Ms4447–4448, and Ms5634–5635. We constructed the recombineering DNA fragments for deletion of Ms4447–4448 and Ms5634–5635 (Supplementary Fig. S2 and Fig. 3b), and then sequentially deleted Ms4447–4448 and Ms5634–5635 from the Ms1277–1278/Ms1283–1284 double-mutant strain (see details in Materials and Methods). The resultant mutant colonies were verified by PCR, yielding the expected PCR products for each of the four mutations with primer pairs p1/p2, p5/p6, p10/p11, and p13/p14 (Fig. 5). The *M. smegmatis* mutant harboring all four mutations was cultured for 100 generations, and then ten colonies were picked and analyzed by PCR. The expected PCR products were obtained in all tested isolates (data not shown), indicating that quadruple-knockout *M. smegmatis* was stable and confirming that the four *dif* sites did not engage in recombination. In addition, the growth rate of the wild type strain and the four mutants were measured. As shown in Supplementary Fig. S3, the mutants showed similar growth rates as the wild type strain, suggesting the presence of *dif* sites on the chromosome of *M. smegmatis* did not affect the bacterial growth. Taken together, these results demonstrate that our novel system can be used to delete multiple genes in *M. smegmatis*.

## Discussion

Gene knockout in mycobacteria is time-consuming, especially if multiple knockouts are needed. In this study, we constructed an improved recombination system for creating multiple unmarked deletions in *M. smegmatis*. Compared to previous recombination systems, our system has several advantages. 1) Following recombination, cells cured of pJV53-GFP could be easily identified (Fig. 2); 2) The Zeo-resistance gene cassette provides a new option for targeted strains that already harbor a Hyg-resistance gene; 3) The Hyg- and Zeo-resistance cassettes can be used in alternation when multiple genes are to be deleted sequentially, by which it can save the time for curing antibiotic gene. However, an additional step is required to cure the antibiotic gene is needed before the next gene can be deleted if only one antibiotic resistance cassette is used.

Flanking *dif* sites can be recognized and resolved by the endogenous recombinases XerC and XerD, leaving a single *dif* site^13^,^14^. Thus, knocking out multiple genes using this system will leave multiple *dif* sites throughout the genomes, raising the possibility that two resolved *dif* sites located near each other could be subjected to another round of recognition and resolution, resulting in deletion of the intervening chromosomal sequence (Fig. 4a,c). This problem can be solved by orienting the *dif* sites of constructs targeting nearby genes in opposite directions (Fig. 4b,c). On the other hand, *dif* sites that are separated by larger chromosomal distances are not resolved, either because they are simply too far apart to be recognized by Xer recombinases or because the presence of essential genes in the intervening chromosomal sequence causes resolution to be a lethal event. In any case, our results suggested that multiple *dif* sites can stably exist on the chromosome of *M. smegmatis*; therefore, multiple deletions can be obtained using this system.

Because our modified plasmids are based on Xer-cise and Che9c recombination systems, they should also be applicable to *M. tuberculosis*, in which these enzymes have been successfully tested. Multiple gene deletion is necessary for the study some genes, for example toxin-antitoxin genes. However, obtaining multiple gene deletion especially in *M. tuberculosis* is a time-consuming work. The new system described in this paper could be useful in such studies because it facilitates the construction of multiple genes knockouts in mycobacteria.

## Methods

### Bacterial strains and culture conditions

*M. smegmatis* mc^2^ 155 was grown in Middlebrook 7H9 broth (Difco) supplemented with 0.05% Tween 80, or on Middlebrook 7H10 agar (Difco) with appropriate antibiotics (25 μg/ml kanamycin, 50 μg/ml hygromycin, or 50 μg/ml zeocin).

### Plasmid construction

The *gfp* gene was amplified by PCR from pSL001^17^ with primers gfpF and gfpR, digested with *Spe*I, and inserted into the corresponding restriction site of pJV53 to yield plasmid pJV53-GFP (Fig. 1a). The *dif-hyg-dif* cassette was synthesized and cloned into vector pUC57 to yield pUC-Hyg (Fig. 1b). The Zeo-resistance gene was amplified by PCR from pSL002^17^ with primers zeoF and zeoR, and cloned into pUC-Hyg to replace the hygromycin resistance gene, yielding pUC-Zeo (Fig. 1c). A 521 bp DNA fragment upstream of Ms1283 and a 568 bp DNA fragment downstream of Ms1284 were amplified by PCR and cloned into pUC-Hyg, yielding pYC710 (Supplementary Fig. S1a). A 513 bp DNA fragment upstream of Ms1277 and a 568 bp DNA fragment downstream of Ms1278 were amplified by PCR and inserted into pUC-Zeo in the same or opposite direction as the Zeo-resistance gene, yielding plasmids pYC711 and pYC772, respectively (Supplementary Fig. S1b and S1c). A 539 bp DNA fragment upstream of Ms4447 and a 548 bp DNA fragment downstream of Ms4448 were amplified by PCR and cloned into the pUC-Hyg to yield pYC738 (Supplementary Fig. 1d). A 587 bp DNA fragment upstream of Ms5635 and a 607 bp DNA fragment downstream of Ms5634 were amplified by PCR and cloned into pUC-Zeo to yield pYC799 (Supplementary Fig. S1a). A 587 bp DNA fragment upstream of Ms5635 and a 613 bp DNA fragment downstream of Ms5634 were amplified by PCR and cloned into pSL001 to yield pYC824. The primers used in this study are listed in Supplementary Table S1.

### Allelic recombination in *M. smegmatis*

Competent *M. smegmatis* mc^2^155 cells containing pJV53-GFP were prepared as previously described^8^. The cells were electroporated with fragments containing the homologous arms and antibiotic cassette, recovered by shaking for 4 h at 37 °C, and plated on 7H10 agar supplemented with kanamycin and a specific antibiotic (hygromycin or zeocin). Hyg- or Zeo-resistant colonies were picked, and streaked on the 7H10 agar and then cells harboring the successful allelic recombination were identified by PCR using appropriate primers. The recombinant cells were plated and grown at 37 °C for 3 days on 7H10 plates supplemented with 10% sucrose. Colonies that lost the GFP signal were identified using a NightSea flashlight and filter glasses, and then tested for the loss of plasmid pJV53-GFP and excision of the dif-antibiotic cassette using PCR and replica streaking on the plate with or without appropriate antibiotics.

### Detailed procedure for sequential deletion of multiple genes in *M. smegmatis*

1. Transform pJV53-GFP into *M. smegmatis* mc^2^ 155 and grow cells overnight with shaking at 37 °C in 7H9 medium supplemented with 25 μg/ml kanamycin.
2. Dilute the resultant culture into 7H9 media containing 0.2% succinate and kanamycin at an OD_600_ of 0.02, and incubate with shaking at 37 °C to an OD_600_ of 0.4–0.5 (~15 hr). Add acetamide to a final concentration of 0.2% (w/v) and grow for another 3 hr.
3. Store competent cells at −80 °C until further use.
4. Construct recombineering *dif-zeo-dif* (or *dif-hyg-dif*) linear DNA fragment by cloning the upstream and downstream flanking regions (~500 bp) of the gene to be deleted into pUC-Zeo (or pUC-Hyg).
5. Digest and recycle the constructed linear DNA fragments by transformation into the competent cells obtained in steps 1–3. Recover the transformants by shaking for 4 hr at 37 °C, and then plate on 7H10 agar supplemented with kanamycin and zeocin or hygromycin as appropriate.
6. Incubate the plate at 37 °C for 3 days. Pick several colonies, streak on 7H10 agar supplemented with kanamycin, and then verify the knockout by PCR using appropriate primers.
7. Pick a verified colony and prepare competent cells as in steps 1–3.
8. Construct a second recombineering linear DNA fragment containing a different antibiotic resistance gene as described in step 4.
9. Transform the second recombineering linear DNA fragment into the competent cells obtained in step 7. Pick several colonies and verify the knockout of the second gene by PCR. In addition, verify the loss of the antibiotic gene used for the first knockout (steps 4–6).
10. Repeat step 7–9 as necessary to delete the third and subsequent genes.
11. Plate the final strain on 7H10 agar supplemented with 10% sucrose, and then grow at 37 °C for 3 days. Pick GFP-negative colonies to verify the loss of pJV53-GFP and excision of the *dif-hyg-dif* or *dif-zeo-dif* cassette.
12. Verify all mutated genes in the resultant isolate by PCR and sequencing.

## Additional Information

**How to cite this article**: Mao, X.-J. *et al.* Efficient and simple generation of multiple unmarked gene deletions in *Mycobacterium smegmatis*. *Sci. Rep.* **6**, 22922; doi: 10.1038/srep22922 (2016).

## Supplementary Material

Supplementary Information
