# Supplementary material for: Efficient and simple generation of multiple unmarked gene deletions in Mycobacterium smegmatis
Source: Sci Rep. 2016 Mar 14;6:22922. doi: 10.1038/srep22922 (PMC4789726; doi:10.1038/srep22922)

Efficient and simple generation of multiple unmarked gene deletions in *Mycobacterium smegmatis*

Xu-Jian Mao^1, 2^, Mei-Yi Yan^1, 2^, Hui Zhu^1^, Xiao-Peng Guo^1^, Yi-Cheng Sun^1^,*

^1^ MOH key laboratory of Systems Biology of Pathogens, Institute of Pathogen Biology, Chinese Academy of Medical Sciences and Peking Union Medical College, 9, Dongdan Santiao, Dongcheng District, Beijing, 100730, China.

^2^ These authors contribute equally to this work.

* To whom correspondence should be addressed. Tel: +86 10 6783 7366; Fax: +86 10 6783 7321; Email: [sunyc@ipbcams.ac.cn](mailto:sunyc@ipbcams.ac.cn)

Table S1. DNA primers used in this study

| Primer name | Sequence 5′–3′ | Restriction enzyme site |
| --- | --- | --- |
| gfpF | ggactagtttgatctgtgcgttcgcacg | SpeI |
| gfpD | ggactagtacgatgacgggctggtgata | SpeI |
| Ms1283F | ggggtaccgaattggcgcagctcgacct | KpnI |
| Ms1283R | cgggatccgtcggcttccgggtgtttga | BamHI |
| Ms1284F | ggactagtcgtgcaggcctgaattacgg | SpeI |
| Ms1284R | cccaagcttccacactggttcgagcatgt | HindIII |
| Ms1277F | gctctagaccacaacggttcgacagga | XbaI |
| Ms1277R | gaagatctgcgtgcgcgaacttcttcagc | BglII |
| Ms1278F | ggactagtgcggaggacctcatgaacga | SpeI |
| Ms1278R | cccaagcttacccaggcgtatgccttctc | HindIII |
| Ms1277RF | cccaagcttgccacaacggttcgacagga | HindIII |
| Ms1277RR | ggactagtcgtgcgcgaacttcttcagc | SpeI |
| Ms1278RF | gaagatctgcggaggacctcatgaacga | BglII |
| Ms1278RR | gctctagaacccaggcgtatgccttctc | XbaI |
| Ms4447F | ggggtacccatcgctgtcgcgaagttcc | KpnI |
| Ms4447R | cgggatccttcgggtgcattcacgtcgg | BamHI |
| Ms4448F | ggactagtacagacatggtcaggaccga | SpeI |
| Ms4448R | cccaagcttgagtggcaccgaacacgatc | HindIII |
| Ms5635F | ggggtaccccaagatcttcggcacggat | KpnI |
| Ms5635R | gctctagagccttgtccaggaatcccat | XbaI |
| Ms5634F | ggactagtctgatcggcaagatcaagcc | SpeI |
| Ms5634R | cccaagctttcgtcatgagcggttactgg | HindIII |
| Ms5635F-pSL001 | ggactagtccaagatcttcggcacggat | SpeI |
| Ms5635R-pSL001 | gccttgtccaggaatcccat |  |
| Ms5634F-pSL001 | agccttaattaagtcaagctgatcggcaagat | PacI |
| Ms5634R-pSL001 | ccaatgcattcgtcatgagcggttactgg | NsiI |
| pUC-hygF | tcttgtgtcacagcggacct |  |
| pUC-hygR | cacgacttcgaggtgttcga |  |
| zeoF | cttaattccgcacggccggttcaccgaggcagttccatag |  |
| zeoR | accgcccccggcgcctgatcgtcggtcagtcctgctcct |  |
| P1 | tgcgattctcgaccgacttc |  |
| P2 | aactgctcgacagcttcccg |  |
| P3 | gaactcgggctacggtccaa |  |
| P4 | tacggggttggtgctctcgc |  |
| P5 | tctggcaatggcatccaaca |  |
| P6 | tcgcgtcgtaatccacgatc |  |
| P7 | tcagggtcgcgagcttgtaa |  |
| P8 | cttggccatggtgaatcctc |  |
| P9 | gaggattcaccatggccaag |  |
| P10 | gaagaactggtccgcaacgt |  |
| P11 | cgccgaatggcatcagaact |  |
| P12 | aagctgatgctcgcgcatgt |  |
| P13 | ttcgtgtcctatctcgtggc |  |
| P14 | cagttacggcaaggtgctca |  |
| P15 | accaagaccgtgtactcggt |  |

Supplementary Figure S1. Diagram of the deletion of Ms1277-1278. dif indicates the *dif* sequence and its direction. The direction of *dif-zeo-dif* was either the same direction (a) or the opposite direction (b). The triangles indicate the primers and their directions. The linear fragments were cut from pYC711 (a) or pYC772, respectively.


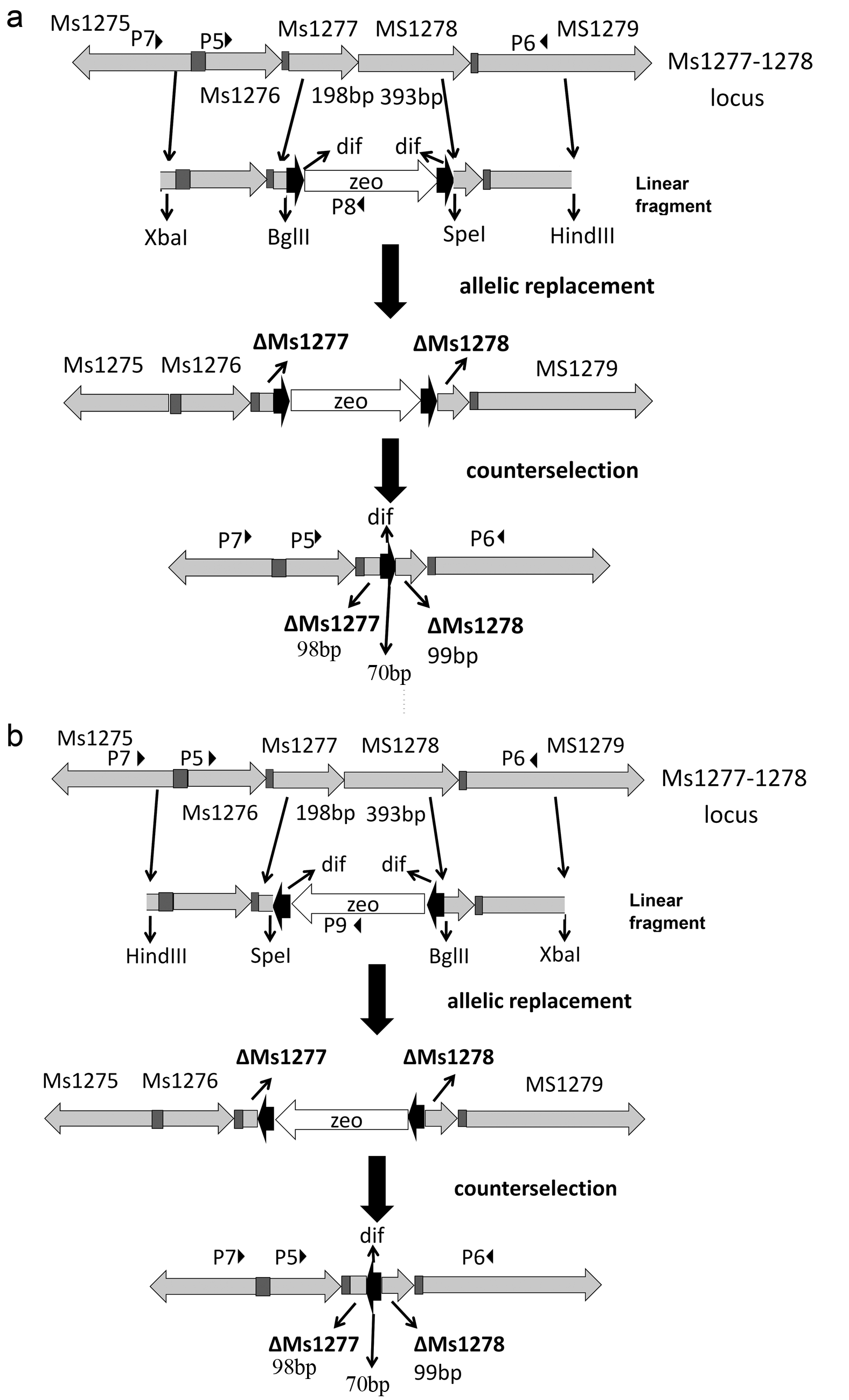


Supplementary Figure S2.

Diagram of the deletion of Ms4447-4448. dif indicates the *dif* sequence and its direction. The triangles indicate the primers and their directions. The linear fragments were cut from pYC38.

**
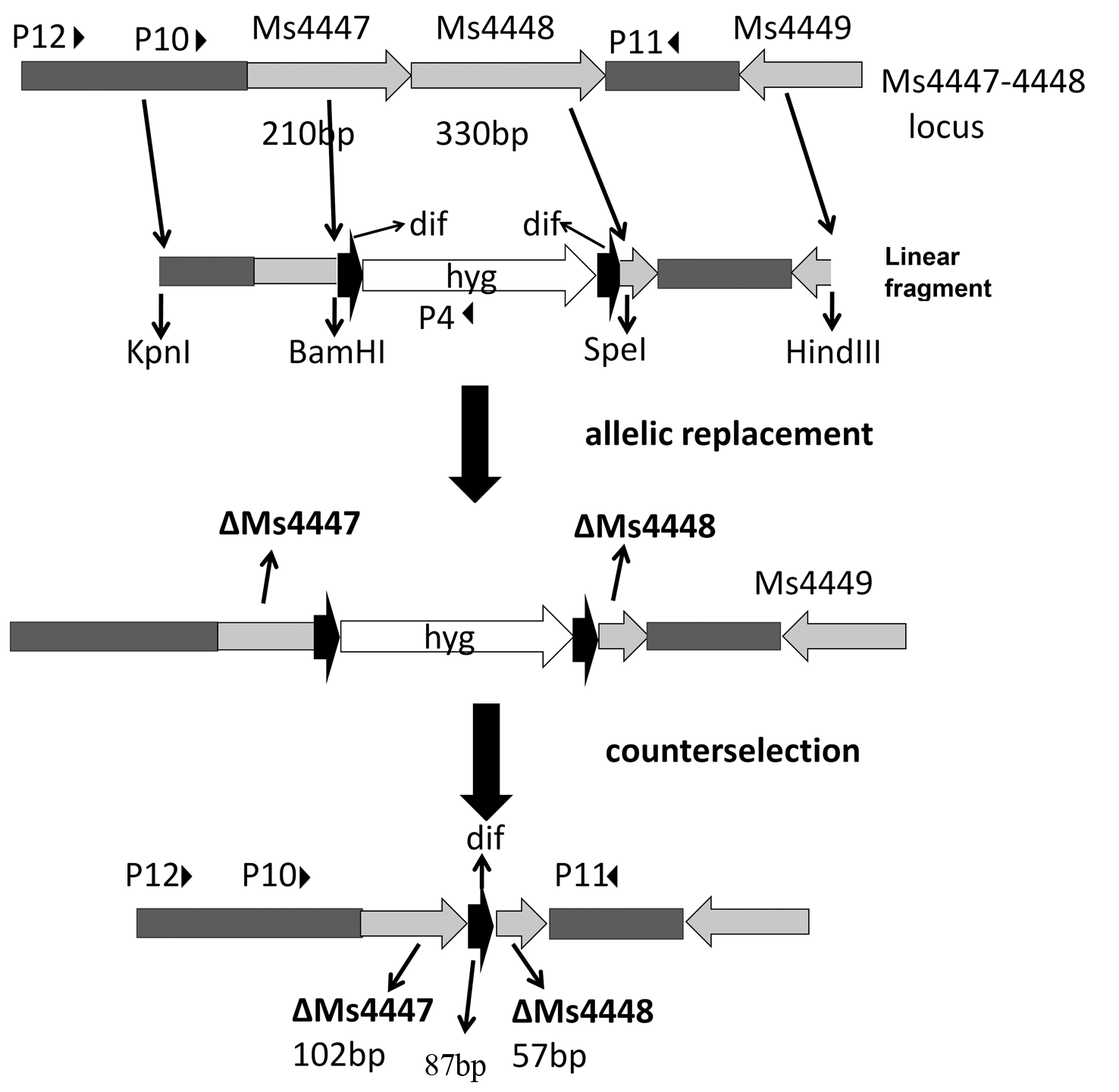
**

Supplementary Figure S3.

Growth of wild type *M. smegmatis* and its derivatives. WT, wild type *M. smegmatis*; M1, *M. smegmatis* with deletion of Ms1283-1284; M2, *M. smegmatis* with deletion of Ms1283-1284 and Ms1277-1278; M3, *M. smegmatis* with deletion of Ms1283-1284, Ms1277-1278 and Ms4447-4448; M4, *M. smegmatis* with deletion of Ms1283-1284 Ms1277-1278, Ms4447-4448 and Ms5634-5635.


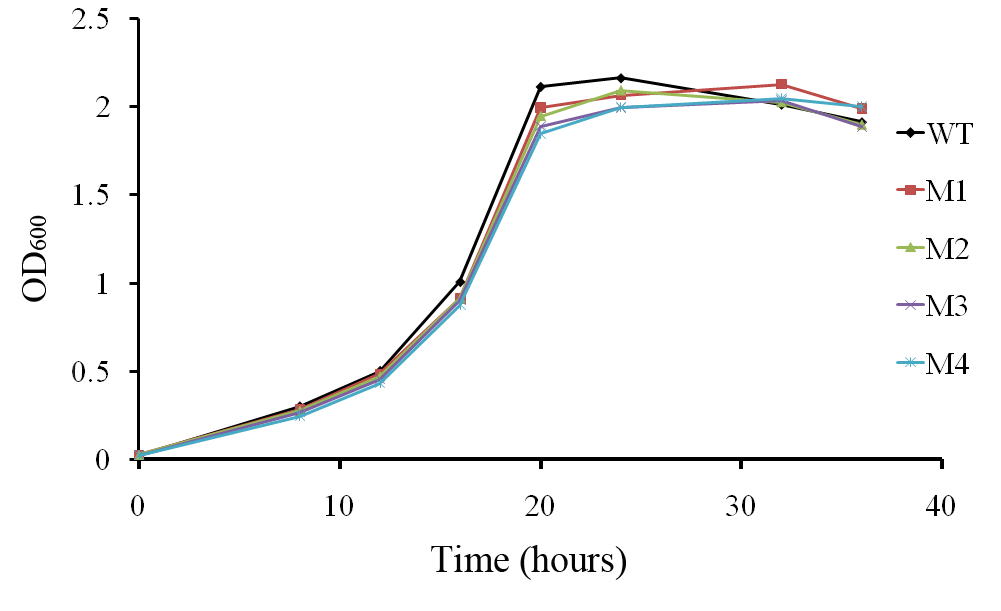

Supplement: Supplementary Information [file srep22922-s1.doc]
